# Supplementary material for: The Impact of Mineral and Energy Supplementation and Phytogenic Compounds on Rumen Microbial Diversity and Nitrogen Utilization in Grazing Beef Cattle
Source: Microorganisms. 2023 Mar 22;11(3):810. doi: 10.3390/microorganisms11030810 (PMC10051884; doi:10.3390/microorganisms11030810)
Supplement: Supplementary file 1 [file microorganisms-11-00810-s001.zip › microorganisms-2280664-supplementary.pdf]

Table S1. Median ± interquartile range of alpha diversity as richness index (ACE and Chao 1) and diversity estimators (Fisher, Simpson, and Shannon) of ruminal microbial population in Nellore cattle grazing Urochloa brizantha cv. Marandu and supplemented or not with phytogenic compounds during the rainy season.

	**Supplements[1]**				**P-value[2]**		
	**EW**	**EPHA**	**MW**	**MPHA**	**ST**	**PHA**	**ST × PHA**
ACE	103.78 ± 45.96	120.11 ± 38.86	89.32 ± 67.53	98.35 ± 39.41	0.363	0.813	0.729
Chao1	100.50 ± 45.75	116.50 ± 39.50	86.50 ± 66.50	95.00 ± 38.00	0.363	0.782	0.723
Fisher	13.35 ± 4.13	15.27 ± 2.92	11.61 ± 6.38	13.27 ± 4.98	0.236	0.937	0.639
Shannon	3.61 ± 0.27	3.59 ± 0.25	3.52 ± 0.40	3.46 ± 0.24	0.144	0.693	0.429
Simpson	0.95 ± 0.012	0.95 ± 0.012	0.95 ± 0.016	0.94 ± 0.011	0.198	0.621	0.498

[1] EW= energy supplement without phytogenic compounds addition. EPHA= energy supplement with phytogenic compounds addition. MW= mineral supplement without phytogenic compounds addition. MPHA = mineral supplement with phytogenic compounds addition. [2] ST = effect of the type of supplement as mineral or energy supplement by a Friedman test. PHA = effect of the addition of phytogenic compounds blend containing 10% of carvacrol and cinnamaldehyde oil and 90% hydrolyzable tannins extracted from berries and grapes at a dose of 1.5 g/kg of ingested dry matter by a Friedman test.
